# Training Children for Prehospital Aquatic Interventions: Assessing Throwing Skills Using Traditional and Alternative Materials in a Simulated Water Rescue

**DOI:** 10.1017/S1049023X24000554

**Published:** 2024-10

**Authors:** Lucía Peixoto-Pino, Roberto Barcala-Furelos, Miguel Lorenzo-Martínez, Adrián Gómez-Silva, Javier Rico-Díaz, Antonio Rodríguez-Núñez

**Affiliations:** 1. Faculty of Education Sciences, University of Santiago de Compostela, A Coruña, Spain; 2. CLINURSID Research Group, Psychiatry, Radiology, Public Health, Nursing and Medicine Department; University of Santiago de Compostela, A Coruña, Spain; 3. REMOSS Research Group, Faculty of Education and Sports Sciences, University of Vigo, Pontevedra, Spain; 4. ESCULCA Knowledge and Educational Action Research Group, University of Santiago de Compostela, A Coruña, Spain; 5. Faculty of Nursing, University of Santiago de Compostela, A Coruña, Spain; 6. Pediatric Critical, Intermediate, and Palliative Care Section, University Clinical Hospital of Santiago de Compostela, A Coruña, Spain; 7. Collaborative Research Network Orientated to Health Results (RICORS): Primary Care Interventions to Prevent Maternal and Child Chronic Diseases of Perinatal and Developmental Origin, RD21/0012/0025, Instituto de Salud Carlos III, Madrid, Spain; 8. Simulation and Intensive Care Unit of Santiago (SICRUS) Research Group, Health Research Institute of Santiago, University Hospital of Santiago de Compostela (CHUS), A Coruña, Spain

**Keywords:** do-it-yourself, drowning, life-saving, PET bottle, rescue tube, ring buoy, safe rescue

## Abstract

**Background:**

Drowning remains a significant cause of mortality among children world-wide, making prevention strategies crucial. The World Health Organization (WHO) recommends training children in safe rescue techniques, including the use of basic skills such as throwing floating objects. This study aims to address a knowledge gap regarding the throwing capabilities of children aged six to twelve using conventional and alternative water rescue materials.

**Method:**

A total of 374 children aged six to twelve years participated in the study, including both males and females. A randomized crossover approach was used to compare throws with conventional rescue material (ring buoy and rescue tube) to an alternative material (polyethylene terephthalate [PET]-bottle). Throwing distance and accuracy were assessed based on age, sex, and the type of rescue tools used.

**Results:**

Children of all ages were able to throw the PET-bottle significantly farther than both the ring buoy (P <.001; d = 1.19) and the rescue tube (P <.001; d = 0.60). There were no significant differences (P = .414) in the percentage of children who managed to throw each object accurately.

**Conclusion:**

Conventional rescue materials, particularly the ring buoy, may not be well-suited for long-distance throws by children. In contrast, lighter and smaller alternatives, such as PET-bottles, prove to be more adaptable to children’s characteristics, enabling them to achieve greater throwing distances. The emphasis on cost-effective and easily accessible alternatives should be implemented in drowning prevention programs or life-saving courses delivered to children.

## Introduction

Children are over-represented in fatal and non-fatal drowning incidents,^1^ and drowning is one of the top five causes of death in over 40 countries up to the age of 14.^1^ In order to reduce world-wide fatal drowning, the World Health Organization (WHO; Geneva, Switzerland) recommends teaching children safe rescue without entering the water^2,3^ by learning and practicing elementary techniques such as throwing floating objects.^3^ For this reason, the concept of “throw, row, and don’t go” has become popular^4,5^ as it is considered one of the paradigms of prevention.^6^

Although there is a lack of empirical research regarding the most effective type of water rescue equipment for a lay responder when assisting a drowning victim, for this reason in 2019, Beale-Tawfeeq, et al posed the key question: What are the most effective types of aquatic rescue equipment for a layperson/bystander to use to rescue a drowning person?^7^

When a person witnesses a drowning, they may feel the instinct or duty to help.^7^ However, this can have fatal consequences.^8^ This phenomenon is referred to as the Aquatic Victim-Instead-of-Rescuer (AVIR) syndrome,^9^ which can occur in both adults^8^ and children.^10,11^ To prevent AVIR syndrome, teaching throwing skills could contribute to reducing the burden of drowning.^2,4,12^ However, there is a knowledge gap that has not yet been addressed regarding the most effective type of water rescue equipment for a lay responder.^13^ How far are individuals able to reach with a throw? Would the throw be accurate? Are conventional rescue materials adapted to the characteristics of children? In the scientific literature, rescue materials such as ring buoys,^14^ rescue tubes,^15^ or do-it-yourself (DIY) materials like polyethylene terephthalate (PET)-bottles^16^ can be found.

The hypothesis of this study is that conventional rescue materials are not suitable for long-distance throws, while lightweight and smaller elements are better suited for children’s characteristics, allowing for greater distances to be reached. Therefore, the aim of this research was to analyze the throwing capacity (distance and accuracy) of two specific rescue materials (ring buoy and rescue tube) and compare them with a non-conventional material, the PET-bottle.

## Methods

### Sample

**Figure 1.**
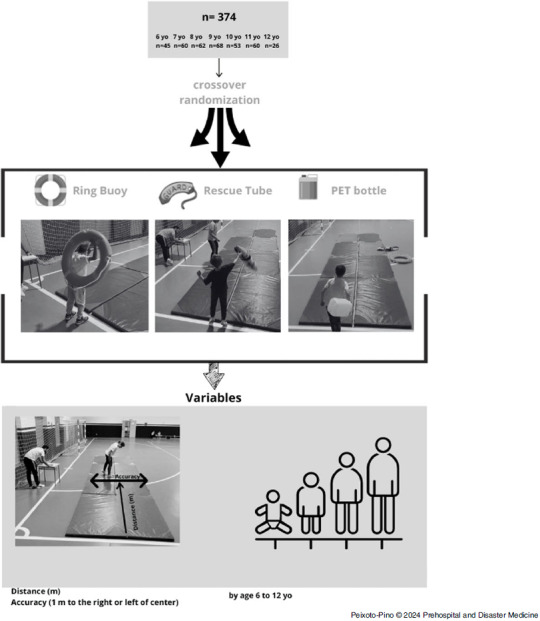
Flow Chart Design. Abbreviation: PET, polyethylene terephthalate.

A total of 374 children (181 males and 193 females) aged six to twelve years (age: 8.8 [SD = 1.8] years; height: 138.0 [SD = 11.7] cm; weight: 33.0 [SD = 8.4] kg) participated in this study. The sample size for each age cohort was as follows: six years: n = 45; seven years: n = 60; eight years: n = 62; nine years: n = 68; ten years: n = 53; eleven years: n = 60; and twelve years: n = 26 (Figure 1). The inclusion criteria required that participants did not have any physical or mental handicaps that would limit their ability to perform the tests. Children who did not meet the inclusion criteria but wished to collaborate were invited to participate in the study, although their data were excluded from the final results. All guardians of participants provided authorization for the use of their data through informed consent. The study received approval from the Ethics Committee of the Faculty of Education and Sports Sciences (University of Vigo, Spain) with the code 07-170123.

### Study Design

A randomized crossover study design was used to examine the differences between conventional rescue equipment, such as a ring buoy and rescue tube, with a PET-bottle. The children participating in the study were presented with the following scenario:

*In a pool, you observe someone drowning. Since you cannot enter the water to avoid endangering your own life, you must throw a floating object to help. Throw it as far and as centered as you can!*

The test dynamics were as follows. Randomly, each child was required to throw each of the three materials from a space simulating a poolside in a dry land scenario, which was previously used in an Australian study.^5^ To ensure that the throw was as realistic as possible, no prior familiarization with the weight or dimensions of the materials was allowed. To prevent learning biases, each test was conducted individually and supervised by two members of the research team.

### Variables

Two types of variables were analyzed: (1) throw distance in meters (m) by age/material, measured from the throw point to the furthest point of the material after the fall, and (2) accuracy, which was defined as the object landing within a maximum of one meter to the left or the right of the center of the linear projection from the throw point (sufficient for it to be reached by stretching an arm). The assessment was recorded by two members of the research team at the end of each test using a tape measure. After the throw, one researcher positioned themselves at the point of initial contact with the material, while the other measured the distance from the throw point to the position indicated by the first.

### Rescue Equipment Characteristics

**Figure 2.**
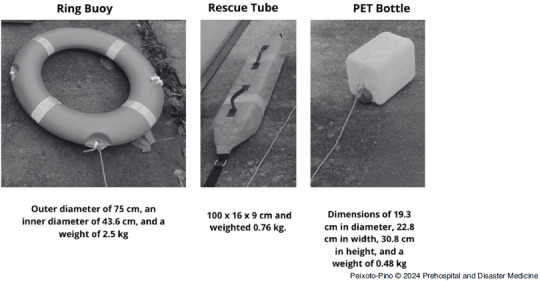
Material Characteristics. Abbreviation: PET, polyethylene terephthalate.

The ring buoy had an outer diameter of 75cm, an inner diameter of 43.6cm, and weighted of 2.5kg. The rescue tube had dimensions of 100cm x 16cm x 9cm and weighted 0.76kg. A 10-liter PET-bottle was used, with dimensions of 19.3cm in diameter, 22.8cm in width, 30.8cm in height, and a weight of 0.48kg (Figure 2).

### Statistical Analyses

All analyses were conducted using the statistical package SPSS for Windows (version 25.0; IBM Corp.; Armonk, New York USA). The normality of each variable was checked both graphically and using the Kolmogorov-Smirnov test. Descriptive statistics for these variables are presented as mean (standard deviation/SD). A repeated-measures analysis of variance (ANOVA) was used to analyze the throwing distance of the subjects according to the equipment used (ring buoy, rescue tube, or PET-bottle). Age and sex of participants were also included in the analysis as inter-subject factors. Partial eta-squared (η^2^_p_) effect sizes were calculated for this analysis. A value η^2^_p_ ≥ 0.01 indicates a small effect, ≥ 0.059 a medium, and ≥ 0.138 a large effect. Pairwise comparisons were conducted using the Bonferroni post-hoc test, with Cohen’s *d* used to calculate the effect sizes. These effects were classified as trivial (*d* < 0.2), small (0.2 < *d* < 0.5), medium (0.5 < *d* < 0.8), and large (*d* ≥ 0.8). The differences in accuracy depending on the object thrown were analyzed using Cochran’s *Q* test. For all analysis, the significance value was set at *P* ≤ .05.

## Results

Table 1 shows the differences in throwing distance and accuracy according to the equipment used and sex. Overall, the results of the repeated-measures ANOVA indicated significant differences by equipment (*F* = 308.803; *P* <.001; η^2^_p_ = 0.462). Children were able to throw the PET-bottle significantly farther than the ring buoy (*P* <.001; *d* = 1.19) and the rescue tube (*P* <.001; *d* = 0.60). Children’s throwing distance using the rescue tube was also significantly greater than with the ring buoy (*P* <.001; *d* = 0.83).

**Table 1.** Differences in Throwing Distance and Accuracy According to Object Used and Sex

	Throwing Distance (M, SD)			Accuracy (%)		
	Ring Buoy	Rescue Tube	PET-Bottle	Ring Buoy	Rescue Tube	PET-Bottle
Males	3.4 (SD = 1.3)^a,b^	4.4 (SD = 1.2)^b,c^	5.8 (SD = 2.4)^a,c^	95.0	91.7	91.7
Females	2.8 (SD = 1.1)^a,b^	3.9 (SD = 1.3)^b,c^	4.6 (SD = 1.7)^a,c^	90.2	88.6	92.2
Total	3.1 (SD = 1.2)^a,b^	4.1 (SD = 1.3)^b,c^	5.2 (SD = 2.2)^a,c^	92.5	90.1	92.0

Abbreviation: PET, polyethylene terephthalate.

a Significant difference (*p*<0.05) with rescue tube.

b Significant difference (*p*<0.05) with PET-bottle.

c Significant difference (*p*<0.05) with ring buoy.

In terms of the interaction between the equipment and sex, small effects were observed (*F* = 10.380; *P* <.001; η^2^_p_ = 0.028). The differences in throwing distance when using the PET-bottle compared to the ring buoy were similar between males (*P* <.001; *d* = 1.26) and females (*P* <.001; *d* = 1.22). However, males obtained greater benefits (*P* <.001; *d* = 0.76) than females (*P* <.001; *d* = 0.46) from throwing the bottle compared to the rescue tube, while the difference in throwing distance with the ring buoy compared to the rescue tube was greater in females (*P* <.001; *d* = 0.91) than in males (*P* <.001; *d* = 0.79).

Regarding throwing accuracy, no significant differences were found in the percentage of subjects who managed to throw each object accurately (*Q* = 1.763; *P* = .414). These results were consistent for both males (*Q* = 2.118; *P* = .357) and females (*Q* = 1.762; *P* = .414).

**Figure 3.**
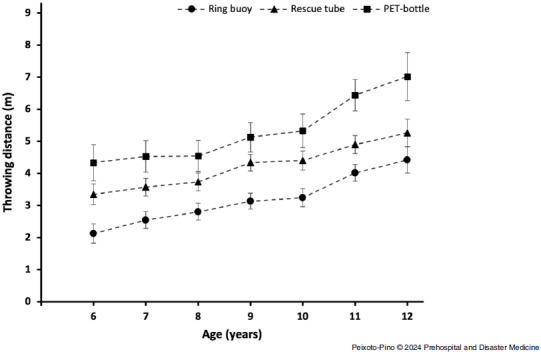
Chart of the Results (Distance/Age). Abbreviation: PET, polyethylene terephthalate.

Table 2 and Figure 3 show the differences in throwing distance depending on equipment and children’s age. The results showed no significant interactions between equipment and age (*F* = 1.464; *P* = .132; η^2^_p_ = 0.024), nor for the interaction between equipment, age, and sex (*F* = 0.765; *P* = .687; η^2^_p_ = 0.013). Regardless of the age, throwing distance with the PET-bottle was always significantly greater than with the ring buoy (*P* <.001; *d* = 1.14–1.70) and the rescue tube (*P* <.01; *d* = 0.53–1.07). Similarly, throwing distance with the rescue tube was also significantly greater than with the ring buoy for all ages (*P* <.001; *d* = 0.65–1.16). Furthermore, throwing distance with all objects tended to increase with age. The largest increases in throwing distance occurred between ten and eleven years old for the ring buoy (*P* = .002; *d* = 0.63) and the PET-bottle (*P* = .050; *d* = 0.52), while for the rescue tube, the largest change occurred between ages eight and nine (*P* = .044; *d* = 0.63).

**Table 2.** Differences in Throwing Distance (M, SD) According to Age

	Age (years)						
	6 [n = 45]	7 [n = 60]	8 [n = 62]	9 [n = 68]	10 [n = 53]	11 [n = 60]	12 [n = 26]
Ring Buoy	2.1 (SD = 0.8)^a,b^	2.6 (SD = 0.9) ^a,b^	2.8 (SD = 1.0) ^a,b^	3.2 (SD = 1.1) ^a,b^	3.2 (SD = 1.1) ^a,b^	4.0 (SD = 1.4) ^a,b^	4.4 (SD = 1.0) ^a,b^
Rescue Tube	3.3 (SD = 1.2)^b,c^	3.6 (SD = 0.9) ^b,c^	3.7 (SD = 1.0) ^b,c^	4.4 (SD = 1.2) ^b,c^	4.4 (SD = 1.0) ^b,c^	4.9 (SD = 1.3) ^b,c^	5.3 (SD = 1.1) ^b,c^
PET-Bottle	4.3 (SD = 1.9)^a,c^	4.6 (SD = 1.8) ^a,c^	4.5 (SD = 1.7) ^a,c^	5.2 (SD = 1.9) ^a,c^	5.3 (SD = 1.9) ^a,c^	6.5 (SD = 2.7) ^a,c^	6.9 (SD = 1.9) ^a,c^

Abbreviation: PET, polyethylene terephthalate.

a Significant difference (*p*<0.05) with Rescue tube.

b Significant difference (*p*<0.05) with PET-bottle.

c Significant difference (*p*<0.05) with Ring buoy.

## Discussion

The aim of this study was to evaluate the distance and accuracy of throws using different materials, including those specifically designed for aquatic rescue and those that are not, with the purpose of aiding a person involved in a simulated drowning scenario. The main findings were as follows: (1) the PET-bottle was thrown accurately and at a greater distance, ranging from four meters at six years old to seven meters at twelve years old; (2) a six-year-old child reached the same distance with a PET-bottle as a twelve-year-old with a ring buoy; and (3) there is a relationship between the weight of the material and the distance it is thrown, with the ring buoy being the material that reaches the shortest distance.

The emphasis on teaching children in safety skills is motivated by their vulnerability to drowning,^17,18^ particularly in middle- and low-resource countries^1,2,19^ where kids are often untrained in aquatic rescues.^20^ Consequently, “non-expert” witnesses should use non-contact rescue techniques without entering the water.^5,6,21^ It is necessary for water safety programs to address two concurrent circumstances in aquatic incidents: the impulse to rescue without analyzing risks^7^ and the fact that when a witness throws an object to save a drowning person, it is often their first time doing so.^5^

To prepare for such scenarios, some institutions advocate achieving a minimum level of competence in throwing aid tools. For instance, the Australian Water Safety Council (AWSC; Broadway, New South Wales, Australia) recommends that children should be capable of throwing a rescue flotation aid to a partner at a distance of five meters before completing primary school (eleven or twelve years old). However, the AWSC report revealed that only a small percentage of children achieved this rescue skill.^22^ For this reason, the current study aimed to identify the age and distance at which children could potentially achieve a life-saving throw.

It was observed that the heavier material (ring buoy) reaches an average distance of two meters at six years old and only increases by an additional two meters throughout primary education, reaching four meters by age twelve. In contrast, the lighter material (PET-bottle) doubles the distance achieved by six-year-olds, practically reaching the same distance (four meters) as preadolescents in the last year of primary education when throwing the ring buoy. At the age of twelve, using the PET-bottle, children can achieve distances similar to those adults throw a lifeline (approximately seven meters).^5^

To achieve an effective throw, two components are needed: strength and coordination. These must be acquired progressively through biological maturation and motor stimulation. Specifically, motor stimulation, as provided in physical education lessons, allows trained children to reach greater distances than adults. Research has shown that individuals under the age of 14 can achieve distances of up to 10 meters when throwing a lifeline.^5^

One fundamental aspect of this research, which is highly practical, is the development of low-cost strategies that can be implemented globally. Modified buoyancy aids can be inexpensive, such as empty plastic containers, bodyboards, or driftwood, and can serve as alternatives to water rescue tools.^15^ The plastic bottle, being the object thrown the farthest, is undoubtedly the most accessible and could be easily adopted by all children as their Rescue-Pet, with an appearance acceptable for rescue equipment.^15^ The creation of DIY materials is itself an efficient pedagogical strategy,^23,24^ and in drowning prevention, teachers in schools form a powerful core for promoting life skills.^25^ The combination of school education with community-wide programs, especially involving parents, can effectively reduce drowning incidents.^26^

## Limitations

This study has limitations that should be noted. Firstly, it involves a local sample without cultural, racial, or socio-cultural diversity. A more diverse sample could yield different results. Additionally, the major limitation is that it is a simulation in which children must imagine being in an aquatic environment and throwing an object to someone who is drowning. Similar to the challenges faced in teaching school-based cardiopulmonary resuscitation/CPR,^27^ further research should analyze the transfer of simulation learning in school to interventions in real-life situations.

## Conclusions

Children can throw any rescue equipment with good accuracy, but at different distances. Conventional rescue materials, particularly the ring buoy, may not be well-suited for long-distance throws by children. In contrast, lighter and smaller alternatives, such as PET-bottles, prove to be more adaptable to children’s characteristics, allowing them to achieve greater throwing distances. The emphasis on cost-effective and easily accessible alternatives underscores the potential for wide-spread implementation in drowning prevention programs.
